# Bibliographical

**Published:** 1883-01

**Authors:** 


### Bibliographical.

*A Guide to the Practical Examination of Urine.* By James Tyson, M. D. Philadelphia: P. Blakiston, Son & Co. 1883.

The fourth edition of this little work does not materially differ from the previous editions. This one contains a rather more exhaustive treatment of several important points concerning urinary analysis, and a few typographical errors are corrected. Though not containing much especially new, the arrangement and the practical hints that the author gives from his large experience make it valuable to busy practitioners who may need a handy and reliable book of reference, and to students preparing for an examination in the subject. Vogel's scale of urine hints is given along with not unfamiliar analysis of urinary calculi.

*Electricity in Medicine and Surgery.* Geo. C. Pitzer, M.D. St. Louis: 1883.

This little work of eighty-three pages does not pretend to deal with the domain of electro-therapeutics in all its various applications. But the object is to furnish the medical student with "the principal facts" of this subject, which to-day is becoming more and more prominent.

The practitioner who has not studied clinically the modus operandi of electrical manipulation will find concise rules for the application of this valuable remedy. Indeed, to one who has employed electricity, the details may seem too simple and too lengthy. But this, to-day is a fault on the right side; and as Dr. Pitzer writes for novices, whether students or physicians who have paid but little attention to this branch, he certainly deserves great praise for his method. The therapeutical part of the work should be enlarged; too large a part now consists in the description of mere apparatus, which, however necessary, is of less importance to the practitioner than therapy. No doubt future editions will remedy this.

*Transactions of the Michigan Dental Society*, at its twenty-seventh annual session, held in Detroit, in March last, are received.

To properly prepare for publication the discussions and debates at such a meeting from the minutes of a stenographer unacquainted with the technicalities of a profession—to present the essence of what every speaker says, no matter how diffuse he may be, nor at what wild tangents he may fly off, to keep the subject matter prominently before the reader despite the wanderings of the speakers—is no easy matter. There are few men who can, without previous preparation, so speak that a verbatim report will clearly express to the reader what the speaker wishes to say. It is the duty of a publication committee to make sense out of the jargon too often submitted to them, to correct the inaccuracies, to harmonize the inconsistencies, and to reduce a mass of seeming chaos to order and sequence, too frequently a task demanding the entire rewriting of long harangues.

The work under notice has been well done. The debates are very fully reported, and the spirit of the themes well preserved.

The Michigan State Dental Society is made up of intelligent and earnest men, and it is little wonder that this, the quarto-centennial anniversary, should find it at a high pitch of prosperity.

*Index-Catalogue of the Library of the Surgeon-General's Office, United States Army. Vol. III. Authors and Subjects. Cholecyanin to Dzondi.* Washington: Government Printing Office, 1882. Quarto. 1,020 pp.

We are in receipt of the third volume of this carefully elaborated catalogue, representing ten thousand and seventy-six volumes, and seven thousand three hundred and eighty-six pamphlets, all catalogued so as to concisely and clearly represent a bibliography of the utmost value to one who wishes to be thoroughly conversant with the literature, past and present, of any one of the (contained) subjects. We notice that medical literature, as exemplified by this library, is especially rich in works concerning cholera, dysentery, diphtheria, diabetes, conjunctivitis, and in those relating to the cranium, in health and disease, with operations thereon.

It is interesting merely to read over the titles of serious essays upon "Epidemics of Demonology," "Human Spontaneous Combustion from Alcoholismus," and "Tea and Coffee in France According to the System of Hippocrates." The labor of compilation must have been immense. The typography and arrangement are most excellent, and the whole book is thoroughly workmanlike.

*Water Analysis. A Handbook for Water-Drinkers.* G. L. AUSTIN, M. D., N. Y. Boston: Lee and Shepard, 1883.

This neat little volume tells the uninitiated how to determine the wholesomeness or unwholesomeness of the drinking water. The test-case containing all the apparatus and reagents necessary is obtainable for $10.

The microscopical examination of drinking water and the sediments therein is summed up in the statement that "animal life in water affords good evidence of the presence of a sensible amount of organic matter or filth." It fails to tell us, however, what to do should we find the water impure, which in the case of Croton water would be the most probable result of an examination.

*Etiology of Dental Caries—Acids or Germs, Which?* This neat pamphlet, reprinted from the *New-England Journal of Dentistry,* is the latest contribution to the study of etiology, and is from the pen of Dr. C. T. Stockwell. In it he urges the theory of the bacterian origin of tooth decay, and presents a very plausible case. Whether accepting or rejecting his conclusions, one cannot fail to admire the zeal with which the author pursues his investigations, and the earnestness with which he presents his views. Right or wrong, such men are the pioneers of progress. The article is a notable contribution to the literature of the subject.

*Importance of Direct Sunlight in the Work-Room.* By J. N. FARRAR, M. D., D. D. S. A reprint from the proceedings of the seventeenth annual meeting of the American Dental Association, in which the influence of the sun's rays upon the bodily health of people engaged in sedentary pursuits is fully considered, and the advisability of a southern exposure for the operating rooms of dentists urged.

*Specialties and their Advantages.* By Dr. Geo. A. Mills. A paper read before the American Dental Convention, at its last annual meeting at Saratoga.

The author urges the necessity for a devotion to a particular field of medical or other sciences, if any great progress is to be made, and cites numerous examples to sustain his views.

*Proceedings of the American Society of Microscopists.* Fifth annual meeting, held at Elmira, N. Y., August, 1882. This is a model report of a memorable meeting. Thirty-two papers were presented, some of them of great interest. We hope to make some extracts from this volume of nearly three hundred pages in a future number.

Pamphlets From Dr. Miller.—We have received from Dr. W. D. Miller, of Berlin, a number of pamphlets and reprints, originally contributed by him to German scientific journals, chiefly *Virchow's Archives*, a translation of some of which we propose at no distant day to present to our readers. As a tireless investigator and a careful observer, Dr. Miller is winning an enviable reputation abroad.

Pamphlets Received.—*Treatment of Arthritis of the Temporo-Maxillary Articulation*, and *The Application by Insufflation of Medicated Powders to the Upper Air-Passages for the Relief of Catarrhal Conditions.* D. H. Goodwillie, M. D., D. D. S. G. P. Putnam's Sons, N. Y., 1882.

